# Prevalence and characteristics of older adults with a persistent death wish without severe illness: a large cross-sectional survey

**DOI:** 10.1186/s12877-020-01735-0

**Published:** 2020-09-17

**Authors:** Iris D. Hartog, Margot L. Zomers, Ghislaine J. M. W. van Thiel, Carlo Leget, Alfred P. E. Sachs, Cuno S. P. M. Uiterwaal, Vera van den Berg, Els van Wijngaarden

**Affiliations:** 1 Department of Care Ethics, University of Humanistic Studies, Utrecht, The Netherlands; 2 Department of Medical Humanities, Julius Center for Health Sciences and Primary Care, University Medical Center, P.O. Box 85060, 3508 AB Utrecht, The Netherlands; 3 Department of Family Medicine, Julius Center for Health Sciences and Primary Care, University Medical Center, Utrecht, The Netherlands; 4 Department of Epidemiology, Julius Center for Health Sciences and Primary Care, University Medical Center, Utrecht, The Netherlands

**Keywords:** Completed life, Tiredness of life, Death wish, Suicide ideation, End-of-life, Euthanasia, Physician-assisted suicide

## Abstract

**Background:**

Some older persons develop a persistent death wish without being severely ill, often referred to as “completed life” or “tiredness of life”. In the Netherlands and Belgium, the question whether these persons should have legal options for euthanasia or physician-assisted suicide (EAS) is intensely debated. Our main aim was to investigate the prevalence and characteristics of older adults with a persistent death wish without severe illness, as the lack of this knowledge is a crucial problem in de debate.

**Methods:**

We conducted a survey among a representative sample of 32,477 Dutch citizens aged 55+, comprising questions about health, existential issues and the nature of the death wish. Descriptive statistics were used to describe the group with a persistent death wish and no severe illness (PDW-NSI) and several subgroups.

**Results:**

A total of 21,294 respondents completed the questionnaire (response rate 65.6%). We identified 267 respondents (1.25%) as having a persistent death wish and no severe illness (PDW-NSI). PDW-NSI did not only occur among the oldest old. Although qualifying themselves as “not severely ill”, those with PDW-NSI reported considerable health problems. A substantial minority of the PDW-NSI-group reported having had a death wish their whole lives. Within the group PDW-NSI 155 (0.73%) respondents had an active death wish, of which 36 (0.17% of the total response) reported a wish to actually end their lives. Thus, a death wish did not always equal a wish to actually end one’s life. Moreover, the death wishes were often ambiguous. For example, almost half of the PDW-NSI-group (49.1%) indicated finding life worthwhile at this moment.

**Conclusions:**

The identified characteristics challenge the dominant “completed life” or “tiredness of life” image of healthy persons over the age of 75 who, overseeing their lives, reasonably decide they would prefer to die. The results also show that death wishes without severe illness are often ambiguous and do not necessarily signify a wish to end one’s life. It is of great importance to acknowledge these nuances and variety in the debate and in clinical practice, to be able to adequately recognize the persons involved and tailor to their needs.

## Background

Improvements in living conditions and health care have contributed to an aging population. Some persons find it difficult to find meaning in older age [1–3]. Some even develop a persistent death wish without being severely ill, which is often referred to as “completed life” or “tiredness of life” [4–6]. “Completed life” is described as “persons, mostly of old age, who do not see a future for themselves and, as a result, have developed a persistent, active death wish, without suffering that (mainly) originates in a medically classifiable condition” [5]. “Tiredness of life” is described as “suffering caused by the prospect of having to continue living with a very poor quality of life, not predominantly caused by a physical or psychiatric disease, and closely associated with a death wish” [6].

In the past decades in the Western world, this death wish is increasingly discussed by the public, encountered by healthcare professionals, and debated in academia, law and politics as a social issue [4, 6–8]. Even though the issue seems to get less attention in countries without legal options for euthanasia or physician-assisted suicide (EAS), the occurrence of persistent death wishes without severe illness seems to be a universal phenomenon. For example, studies from China and Brazil demonstrate its occurrence in different continents and cultural settings [9, 10].

In the Netherlands and (although to a lesser extent) in Belgium, the issue has become highly political. The debate currently centers on the question whether older persons who consider their lives to be “completed” or who are “tired of life” should have legal options for EAS. In the case of euthanasia, a physician administers a lethal substance to terminate the life of a patient at the patient’s own request. Physician-assisted suicide means that a patient at his or her own request takes a lethal substance in the presence of and supplied by a physician [11]. A recent survey indicated that 51% of Dutch citizens are in favor of allowing the oldest old to obtain lethal prescription drugs at their own request from a physician to end their own lives [12].

Dutch physicians are allowed to grant a patient’s request for EAS when six due care criteria are met, including the conviction that the patient is suffering unbearably. However, EAS for “completed life” or “tiredness of life” is not allowed because the suffering of these patients does not predominantly originate from a medical condition, either somatic or psychiatric [11–14]. Citizens and politicians have protested against this restriction, putting the issue high on the political agenda. In 2016, a committee established by the Dutch government explored the legal possibilities and societal dilemmas with regard to assisted dying in cases of “completed life” or “tiredness of life” in old age. It advised against changing the Euthanasia Act [5]. The question how society should respond to the needs and wishes of this particular group of older persons is, however, still intensely debated.

A crucial and under-addressed problem in the debate is the lack of robust knowledge on the prevalence of persons with “completed life” or “tiredness of life” and on their characteristics, existential issues and the nature of their death wishes [14, 15]. Moreover, insight into these aspects may be of great value to healthcare professionals who are increasingly confronted with older persons who are not severely ill but consider their lives not worth living [6]. Recent research among nurses indicated how challenging encounters with these persons can be, for instance in terms of recognizing what is going on [6]. Our objective was to provide the knowledge that is needed to further the debate as well as providing healthcare professionals clues that enable them to better recognize cases of “completed life” or “tiredness of life”. To this end, we aimed to investigate the prevalence of older adults with a persistent death wish without severe illness and their characteristics, existential issues and the nature of their death wishes.

## Methods

### Study design and sample size

We conducted a web-based survey among a representative sample of 32,477 Dutch citizens aged 55 and older. The sample was taken from the access panel “TNS NIPObase” of research company Kantar Public, a leading provider of data for policy making around the world [16]. The panel comprises 109,642 Dutch respondents including 44,667 persons of 55 years and older. The sample was drawn to reflect the known proportions of age, gender, education attainment, household size, social class and region in the Dutch population aged 55 and older. The societal and political debate in the Netherlands indicates that every age limit for research into our target phenomenon would be arbitrary and perhaps even controversial. For example, proponents of legal options for EAS for older persons who consider their lives to be “completed” or who are “tired of life” suggest different age limits, and every proposed age limit is criticized. Reasons for this criticism are, among others, that also younger people might have a wish to die without being severely ill, and that an age limit may unintentionally suggest that life beyond that age is considered not or less worth living anymore. We chose the age limit of 55 and older for two reasons. Firstly, previous research suggests an increase of the prevalence of death wishes with age [17, 18]. Setting the age limit relatively low, we were able to verify this hypothesis in our sample. Secondly, according to psychological literature, from the age of 55, people increasingly draw up the balance of their own lives, and ask themselves to what degree they have accomplished their life goals [19].

### Demarcation of the group of interest

We first demarcated the group of interest, since no clear definitions or operationalizations of “completed life” or “tiredness of life” existed. Currently, the dominant description regarding the health of this group is: “without suffering that (mainly) originates in a medically classifiable condition” or “suffering not predominantly caused by a physical or psychiatric disease”, respectively [5, 6]. As a large survey including self-reported health measures is the appropriate method to estimate prevalence, we chose to rephrase the description of “completed life” or “tiredness of life” referring to people’s own perceptions (i.e. “illness”) rather than diagnosed conditions (professional’s judgments, i.e. “disease”) [20]. Persons perceiving themselves as “not severely ill” are likely not to be eligible for EAS, which is only allowed in cases of unbearable suffering (mainly) originating in a medically classifiable condition. Therefore, our group of interest was defined as “older adults with a persistent death wish and no severe illness” (PDW-NSI).

### Questionnaire

As no validated or accepted questionnaires existed to identify older adults with persistent death wishes without being severely ill, a questionnaire was developed specifically for this study. It comprised questions about physical and mental health, existential issues and, if applicable, about the nature of the death wish. See Additional file 1 for all the questionnaire items reported in this study. In order to ensure the safety and well-being of the persons in our sample, the questionnaire items about death wishes and suicide attempts were discussed with the research company and the national suicide prevention organization of the Netherlands. As research indicates that including suicidal persons in research or asking questions about suicidality does not increase suicidality [21], these questionnaire items were not considered harmful. The questionnaire introduction and items were carefully built up to prepare respondents for the questions about death wishes, the voluntary character was emphasized and contact details of our research team and the suicide prevention organization in the questionnaire were provided.

To narrow down the sample we asked respondents a “differentiation question” based on our definition of our group of interest mentioned earlier: “Do the qualifications ‘seeing no future for yourself, longing for death while not being severely ill’ apply to you at this moment?”. If they answered affirmatively, the respondents were asked in-depth questions about health, existential issues and the nature of the death wish. For all others, the questionnaire ended, except for what we call the “comparison group”: the respondents from an additional random sample who completed the whole questionnaire despite their negative response to the differentiation question.

EQ-5D-5L (Dutch version) was used to assess health state [22]. This self-report questionnaire comprises five dimensions: mobility, self-care, usual activities, pain/discomfort and anxiety/depression.

HADS-D, the depression subscale of the Hospital and Anxiety Depression Scale (Dutch version), was included to assess the probability of depressive disorder [23]. Due to a logistical error, the last of the seven items of the HADS-D was not included in the questionnaire. Therefore, in a second round, an additional request to complete the whole HADS-D was sent.

### Data analysis

Starting with the respondents who answered the differentiation question with “yes”, we performed a selection in three steps to identify the PDW-NSI group. We used additional measures of health (step 1), depression (step 2), and the self-reported duration of respondents’ death wishes (step 3), to determine the presence or absence of severe illness and the persistence of the death wish.

To identify the group without severe illness (step 1 and 2), validated self-reported measures of overall health and depressive feelings were used. These self-reports are assumed to be the best indication for the possible role of health problems in evaluating one’s own quality of life and in developing a death wish. Conservative cut-off points were chosen to only exclude respondents with severe health problems and an indication for severe depression, in order to avoid exclusion of respondents with health problems who would nevertheless be denied EAS because they do not suffer unbearably from a medically classifiable condition.

The three steps are described below. See Additional file 2 for further details. For a visual representation of the steps, see the Results section.

#### Step 1 – no severe illness: self-reported health

Respondents were categorized as “not severely ill” if they scored ≥ 4 on an 11-point visual analogue scale (VAS) *and* < 17 on the EQ-5D-5L (sum score).

#### Step 2 - no severe illness: indication for depression

Following clinical practice, HADS-D sum scores < 16 were used to categorize the respondents with “no indication for severe depression” [24].

#### Step 3 - persistence of the death wish

In compliance with literature about death wishes and suicidal feelings, we considered a death wish with a duration of ≥ 1 year as “persistent” [25].

After this selection process, the group PDW-NSI was divided into subgroups of having an *active* or a *passive* persistent death wish. Based on literature about death wishes and suicidal ideation, the death wishes of respondents were considered “active” if respondents indicated having made concrete plans or having taken steps regarding their death wish [14, 26]. Respondents who had seriously *considered* attempting suicide in the past 12 months were also regarded as having an active death wish [25, 26]. Respondents reporting no steps/plans and not having considered suicide were categorized as having a passive death wish. The group of respondents who reported no plans or steps and who chose the option “not willing to answer” for the question whether they had considered suicide, were categorized as “passive/active nature of death wish unknown”.

As our response sample did not exactly represent the Dutch population, the prevalence calculations were repeated weighted for gender, age, educational attainment, household size, social class and region.

All calculations were performed using SPSS software, version 25.0 (2018). Confidence intervals were calculated using the Wilson method. Significance tests in group comparisons were calculated using the Kruskal-Wallis test for ordinal and Fisher’s exact test for nominal variables. All prevalence calculations were checked by a biostatistician.

## Results

### Prevalence

**Fig. 1:**
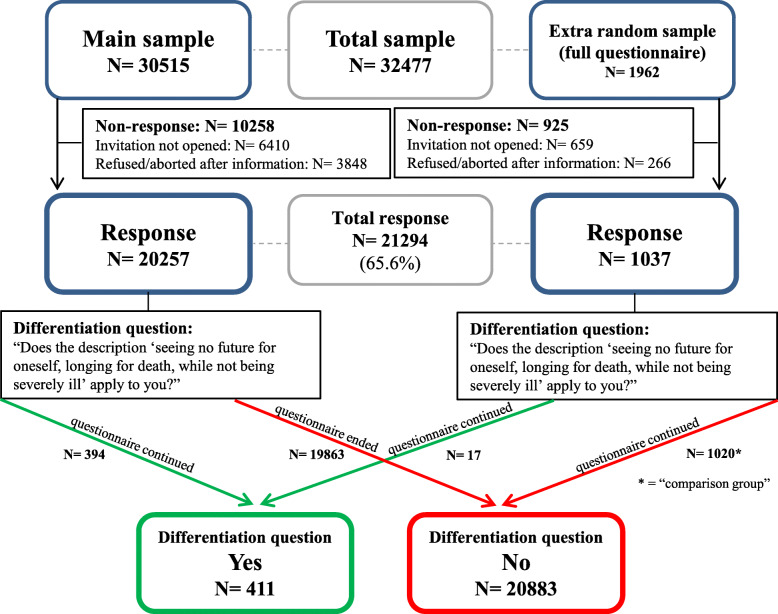
Flowchart sample and response

A total of 21,294 respondents (65.6%) completed the questionnaire between April 3 and April 25, 2019. See Fig. 1 for a flow chart of the sample and response, in which two separate flows are presented. The left side shows the main sample of participants who only completed the whole questionnaire if they answered the differentiation question affirmatively. The right side shows the extra random sample of participants who completed the whole questionnaire regardless of their answer to the differentiation question.

The additional request to complete the whole HADS-D was sent to those with “yes” to the differentiation question (*N* = 411) and those in the comparison group (*N* = 1020) and filled out by 89.2% of these respondents between May 23 and June 3, 2019.

Background characteristics of respondents versus non-respondents are presented in Additional file 3. Differences between respondents and non-respondents were found on all background characteristics except urbanization.

**Fig. 2:**
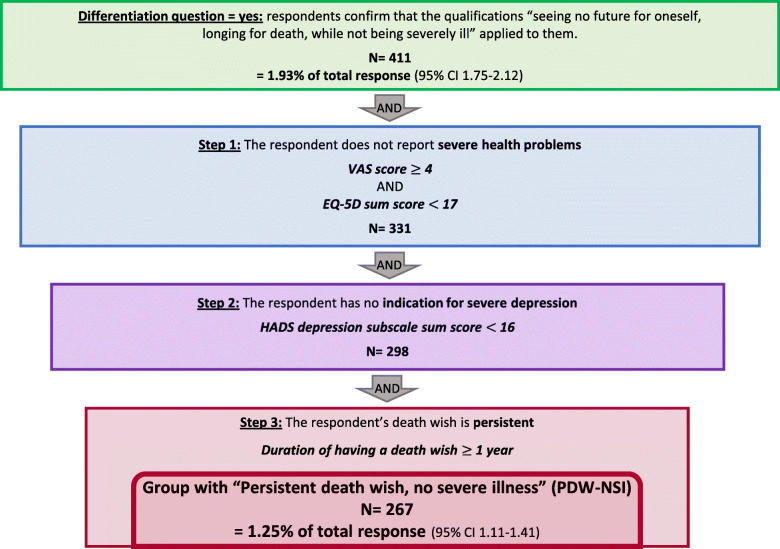
Flowchart selection process to identify the group “persistent death wish, no severe illness” (PDW-NSI). Total response: *N* = 21,294. Percentages may not add up to total because of rounding

Figure 2 shows the steps in the selection process of the PDW-NSI group. Four hundred eleven respondents (1.93%) answered “yes” to the differentiation question. The subsequent selection steps led to the identification of 267 respondents (1.25%) with a persistent death wish without severe illness (PDW-NSI).

**Fig. 3:**
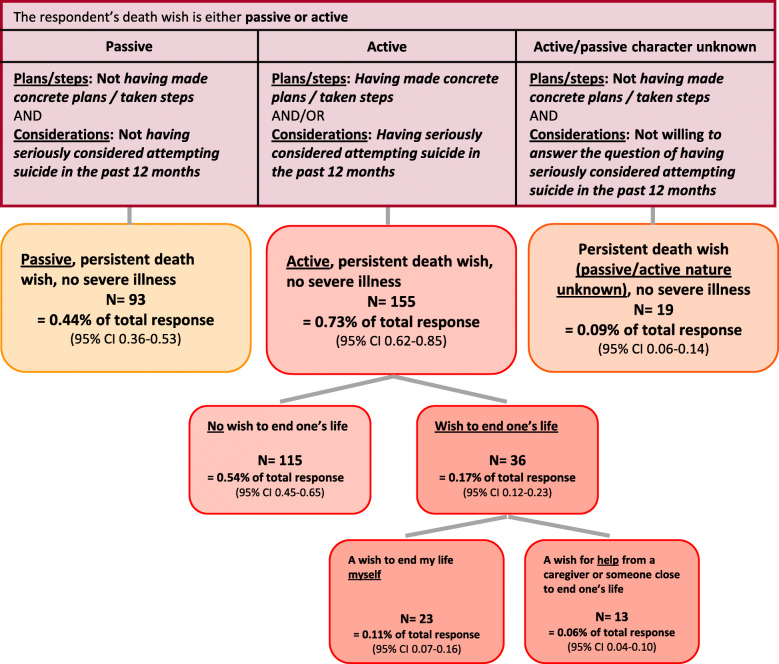
Flowchart selection of subgroups of “persistent death wish, no severe illness” (PDW-NSI). Total response: *N* = 21,294. Percentages may not add up to total because of rounding. Numbers of respondents with and without wish to end their life do not add up to *N* = 155 because 4 respondents answered “I do not know”

Figure 3 shows the categorization of the group PDW-NSI into subgroups. Of the respondents with a persistent death wish without severe illness, 93 (0.44%) had a passive death wish, 155 (0.73%) had an active death wish, and 19 (0.09%) had a death wish of an unknown (passive/active) nature. Of the group with an active persistent death wish, 36 respondents (0.17% of the total response) described their wish as a wish to end their lives.

### Weighted percentages

Weighted percentages representing the Dutch population were 1.34% (95% CI 1.20–1.51) for the entire group PDW-NSI, 0.47% (95% CI 0.38–0.57) for the subgroup with a passive death wish, 0.77% (95% CI 0.66–0.90) for the subgroup with an active death wish and 0.18% (95% CI 0.13–0.25) for the subgroup with an active death wish resulting in a wish to actually end their lives.

### Background characteristics

Table 1 shows background characteristics of the PDW-NSI group and of the respondents who answered “no” when asked the differentiation question whether the qualifications “seeing no future for oneself, longing for death while not being severely ill” applied to them at that moment.

**Table 1:** Background characteristics

Aspects and items	Active persistent death wish, no severe illness ***N*** = 155 (0.7% of 21,294)	Passive persistent death wish, no severe illness ***N*** = 93 (0.4% of 21,294)	Persistent death wish (passive/active nature unknown), no severe illness ***N*** = 19 (< 0.1% of 21,294)	Total group “Persistent death wish, no severe illness” (PDW-NSI) ***N*** = 267 (1.25% of 21,294)	Answer “No” to differentiation question: Does the description “seeing no future for oneself, longing for death, while not being severely ill” apply to you? ***N*** = 20,883 (97.8% of 21,294)	***P***-value PDW-NSI group vs. Diff. question = No	***P***-value Active vs. Passive
**Gender**							
**Female**	87 (56.1)	50 (53.8)	12 (63.2)	149 (55.8)	10,500 (50.3)	0.074	0.792
**Male**	68 (43.9)	43 (46.2)	7 (36.8)	118 (44.2)	10,383 (49.7)		
**Age (years)**							
**Median (Q1-Q3)**	64 (60–72)	67 (60.5–74)	68 (61–75)	65 (60–73)	65 (60–72)	0.161	0.267
**55–59**	37 (23.9)	18 (19.4)	3 (15.8)	58 (21.7)	5119 (24.5)		
**60–64**	44 (28.4)	19 (20.4)	3 (15.8)	66 (24.7)	4569 (21.9)		
**65–69**	21 (13.5)	19 (20.4)	6 (31.6)	46 (17.2)	4156 (19.9)		
**70–74**	23 (14.8)	16 (17.2)	2 (10.5)	41 (15.4)	3977 (19.0)		
**75–79**	16 (10.3)	12 (12.9)	4 (21.1)	32 (12.0)	1796 (8.6)		
**80–84**	8 (5.2)	6 (6.5)	0 (0.0)	14 (5.2)	890 (4.3)		
**85–89**	4 (2.6)	3 (3.2)	1 (5.3)	8 (3.0)	320 (1.5)		
**90–94**	2 (1.3)	0 (0.0)	0 (0.0)	2 (0.7)	51 (0.2)		
**95–99**	0 (0.0)	0 (0.0)	0 (0.0)	0 (0.0)	5 (< 0.1)		
**Educational attainment**^**a**^							
**Low**	54 (34.8)	37 (39.8)	7 (36.8)	98 (36.7)	7018 (33.6)	0.288^b^	0.507^c^
**Middle**	59 (38.1)	27 (29.0)	11 (57.9)	97 (36.3)	7554 (36.2)		
**High**	41 (26.5)	27 (29.0)	1 (5.3)	69 (25.8)	6213 (29.8)		
**Unknown**	1 (0.6)	2 (2.2)	0 (0.0)	3 (1.1)	98 (0.5)		
**Worldview**^**d**^							
**Religious worldview**	52 (31.9)	45 (45.0)	9 (45.0)	106 (37.5)	517 (49.1)	**0**.**002**	**0**.**023**
**Non-religious worldview**	49 (30.1)	27 (27.0)	5 (25.0)	81 (28.6)	200 (19.0)	**0**.**000**	0.776
**Worldview, religiousness unknown**	8 (4.9)	3 (3.0)	0 (0.0)	11 (3.9)	44 (4.2)	1.000	0.544
**No worldview**	54 (33.1)	25 (25.0)	6 (30.0)	85 (30.0)	293 (27.8)	0.327	0.208
**Number of children**^**e**^							
**0**	31 (26.5)	20 (26.7)	5 (35.7)	56 (27.2)	124 (16.3)	**0**.**000**	0.734
**1**	14 (12.0)	14 (18.7)	1 (7.1)	29 (14.1)	96 (12.6)		
**2**	56 (47.9)	29 (38.7)	7 (50.0)	92 (44.7)	329 (43.1)		
**3 or more**	16 (13.7)	12 (16.0)	1 (7.1)	29 (14.1)	214 (28.0)		
**Household size (number of persons)**							
**1**	84 (54.2)	46 (49.5)	9 (47.4)	139 (52.1)	5259 (25.2)	**0**.**000**	0.504
**2**	58 (37.4)	39 (41.9)	10 (52.6)	107 (40.1)	12,480 (59.8)		
**3 or more**	13 (8.4)	8 (8.6)	0 (0.0)	21 (7.9)	3144 (15.1)		
**Social class**^**f**^							
**Low**	78 (50.3)	49 (52.7)	15 (78.9)	142 (53.2)	8406 (40.3)	**0**.**000**	0.934
**Middle**	26 (16.8)	12 (12.9)	0 (0.0)	38 (14.2)	3171 (15.2)		
**High**	51 (32.9)	32 (34.4)	4 (21.1)	87 (32.6)	9306 (44.6)		
**Urbanization**^**g**^							
**Very high**	39 (25.2)	35 (37.6)	3 (15.8)	77 (28.8)	3829 (18.3)^h^	**0**.**001**	0.187
**High**	52 (33.5)	23 (24.7)	8 (42.1)	83 (31.1)	7290 (34.9)		
**Moderate**	26 (16.8)	15 (16.1)	4 (21.1)	45 (16.9)	3813 (18.3)		
**Low**	28 (18.1)	12 (12.9)	3 (15.8)	43 (16.1)	4223 (20.2)		
**None**	10 (6.5)	8 (8.6)	1 (5.3)	19 (7.1)	1722 (8.2)		

Results are presented as N (%) unless “Median (Q1-Q3)” is reported

Percentages may not add up to 100% because of rounding

Medians are reported with 25th–75th percentiles

N/A = not applicable

Statistically significant results (*p* < 0.05) are in bold

^a^Low = lower vocational education, lower secondary education, or less; Middle = intermediate vocational education or higher secondary education; High = higher vocational education or university; Unknown = I do not know/want to answer

^b^*N* = 21,049 because category “Unknown” was not included in the test

^c^*N* = 245 because category “Unknown” was not included in the test

^d^Religious worldview = Protestant, Catholic, Muslim, Jewish, Hindu and Buddhist. Non-religious worldview = atheist, agnostic, “spiritual but not religious”, humanist, anthroposophical and esoteric. Worldview, religiousness unknown = other worldview. Respondents could give more than one answer and may thus be counted in more than one category. Therefore, *N* = 163, *N* = 100, *N* = 20, *N* = 283, *N* = 1054 (comparison group) respectively and percentages are based on these numbers. In group comparisons, worldview is tested with separate tests for each category (yes/no)

^e^*N* = 117, *N* = 75, *N* = 14, *N* = 206, *N* = 763 (comparison group) respectively for the groups, due to missing values for “number of children”

^f^Based on educational attainment and profession of the main breadwinner

^g^Very high= > 2500; High = 1500–2500; Moderate = 1000–1500; Low = 500–1000; None= < 500 addresses per km^2^

^h^*N* = 20,877 in this column due to 6 missings for this variable

Of the PDW-NSI group, 79,0% were under the age of 75. Even though percentages for the age categories above 75 were slightly higher for the PDW-NSI group than for the group who gave a negative answer to the differentiation question, there was no significant overall difference in age distribution. Approximately half of the PDW-NSI group (52.1%) lived alone and 27.2% had no children, both higher proportions as compared to the group with a negative answer to the differentiation question (25.2 and 16.3% respectively). Persons in the PDW-NSI group were of lower social class, lived in highly urbanized areas more often, and a significantly smaller percentage had a religious worldview.

Compared to the subgroup with an active death wish, a larger proportion of the group with a passive death wish had a religious worldview. No significant differences between the subgroups were found for the other background characteristics.

### Health/illness

The PDW-NSI group had qualified themselves as “not severely ill” through their affirmative answer to the differentiation question. Nonetheless, Table 2 shows that all measurements for health and illness indicated significantly worse health for the PDW-NSI group as compared to the group that gave a negative answer to the differentiation question, with the exception of the presence of a life-threatening disease now or in the past. 9.4% of the group PDW-NSI reported having none of the diseases listed, against 28.2% of the group who answered “no” to the differentiation question. Of the respondents with at least one of the diseases listed, those in the group PDW-NSI reported a significantly higher total burden of these diseases (median 7 versus 4 on a scale from 1 to 10). In the group PDW-NSI, 50% of the respondents had a HADS-D sum score between 6.7 and 12, and 25% a sum score of 12 or higher on a scale from 0 to 21, with a cut-off point of 16 for severe depression. The group with answer “no” to the differentiation question reported significantly lower levels of depressive feelings: 50% had sum scores between 1 and 5.

**Table 2:** Health/illness

Aspects and items	Active persistent death wish, no severe illness ***N*** = 155 (0.7% of 21,294)	Passive persistent death wish, no severe illness ***N*** = 93 (0.4% of 21,294)	Persistent death wish (passive/ active nature unknown), no severe illness ***N*** = 19 (< 0.1% of 21,294)	Total group “Persistent death wish, no severe illness” (PDW-NSI) ***N*** = 267 (1.25% of 21,294)	Answer “No” to differentiation question - Does the description “seeing no future for oneself, longing for death, while not being severely ill” apply to you? ***N*** = 20,883 (97.8% of 21,294)	***P***-value PDW-NSI group vs. Diff. question = No	***P***-value Active vs. Passive
**Current health state VAS**^**a**^							
**Median (Q1-Q3)**	6 (5–7)	7 (6–8)	6 (6–7)	6 (6–7)	8 (7–8)	**0**.**000**	**0**.**010**
**EQ-5D-5L sum score**^**b**^							
**Median (Q1-Q3)**	10 (8–13)	8 (7–11)	11 (8–13)	10 (8–12)	7 (5–9)	**0**.**000**	**0**.**000**
**HADS depression subscale, sum score**^**c**^							
**Median (Q1-Q3)**	11 (8–12)	8 (6–11.5)	10 (7–14)	10 (6.7–12)	3 (1–5)^d^	**0**.**000**	**0**.**001**
**Life-threatening disease**							
**Never**	110 (71.0)	76 (81.7)	13 (68.4)	199 (74.5)	16,388 (78.5)	0.196	0.139
**Yes, but not anymore**	33 (21.3)	14 (15.1)	5 (26.3)	52 (19.5)	3595 (17.2)		
**Yes, at this moment**	12 (7.7)	3 (3.2)	1 (5.3)	16 (6.0)	900 (4.3)		
**Number of current diseases**^**e**^							
**None**	9 (5.8)	16 (17.2)	0 (0.0)	25 (9.4)	288 (28.2)^f^	**0**.**000**	**0**.**008**
**Median (Q1-Q3)**	2 (1–4)	1 (1–2)	3 (2–4)	2 (1–3)	1 (0–2)	**0**.**000**	**0**.**000**
**Burden of current diseases**^**g**^							
**Median (Q1-Q3)**	7 (5–8)	6 (4.5–7)	6 (5–8)	7 (5–8)	4 (3–6)	**0**.**000**	**0**.**005**
**Number of current complaints**^**h**^							
**None**	3 (1.9)	5 (5.4)	0 (0.0)	8 (3.0)	195 (19.1)^i^	**0**.**000**	0.155
**Median (Q1-Q3)**	5 (3–7)	3 (2–6)	6 (4–9)	5 (3–7)	2 (1–4)	**0**.**000**	**0**.**000**
**Burden of current complaints**^**j**^							
**Median (Q1-Q3)**	7 (5–7)	5.5 (4–7)	6 (5–8)	6 (5–7)	5 (3–6)	**0**.**000**	**0**.**000**

Results are presented as N (%) unless “Median (Q1-Q3)” is reported

Percentages may not add up to 100% because of rounding

Medians are reported with 25th–75th percentiles

Statistically significant results (*p* < 0.05) are in bold

^a^Visual Analogue Scale ranging from 0 (worst imaginable) to 10 (best imaginable health state)

^b^EQ-5D-5L sum scores range from 5 to 25. Higher sum scores indicate more severe problems on the five domains of health

^c^HADS depression subscale, sum scores range from 0 to 21. Higher sum scores are related to a possible indication for depression with a greater severity

^d^*N* = 1020 (comparison group)

^e^Chosen from the following list of diseases: joint conditions, osteoporosis, diabetes, neck or back problems, tightness of the chest (e.g. COPD, asthma), Crohn’s disease, MS/ALS, skin disease, thyroid disease, heart conditions, consequences of CVA, dementia, Parkinson’s disease, cancer, psychological complaints (mood or anxiety complaints, depression), other

^f^Percentages are based on *N* = 1020 (comparison group)

^g^10-point scale ranging from 1 (“very little”) to 10 (“very much”). Diseases are listed above

^h^Chosen from a list of complaints, such as hearing problems or deafness, tinnitus, headache, dizziness, obesity, incontinence, chronic itching, extreme/chronic fatigue, depressive feelings, obstipation and (chronic) pain, other

^i^Percentages are based on *N* = 1020 (comparison group)

^j^10-point scale ranging from 1 (“very little”) to 10 (“very much”). Complaints are listed above

Respondents with an active death wish had scores indicating worse mental, physical and overall health than the group with a passive death wish.

### Death wishes and existential issues

Table 3 provides insight into the death wishes of respondents with PDW-NSI, and responses on existential issues of both this group and the group with a negative answer to the differentiation question.

**Table 3:** Death wishes and existential issues

Aspects and items	Active persistent death wish, no severe illness ***N*** = 155 (0.7% of 21,294)	Passive persistent death wish, no severe illness ***N*** = 93 (0.4% of 21,294)	Persistent death wish (passive/ active nature unknown), no severe illness ***N*** = 19 (< 0.1% of 21,294)	Total group “Persistent death wish, no severe illness” (PDW-NSI) ***N*** = 267 (1.25% of 21,294)	Answer “No” to differentiation question - Does the description “seeing no future for oneself, longing for death, while not being severely ill” apply to you? ***N*** = 20,883 (97.8% of 21,294)	***P***-value PDW-NSI group vs. Diff. question = No	***P***-value Active vs. Passive
**Duration of having a death wish**							
**Whole life**	27 (17.4)	21 (22.6)	2 (10.5)	50 (18.7)	N/A	N/A	0.481
**A few years**	97 (62.6)	54 (58.1)	17 (89.5)	168 (62.9)	N/A		
**Approximately one year**	31 (20.0)	18 (19.4)	0 (0.0)	49 (18.4)	N/A		
**Characterization of the death wish**							
**A desire for a natural death that just happens**	22 (14.2)	25 (26.9)	3 (15.8)	50 (18.7)	N/A	N/A	**0**.**008**
**A desire to not wake up tomorrow and die in my sleep**	86 (55.5)	43 (46.2)	9 (47.4)	138 (51.7)	N/A		
**I feel my current situation is unlivable**	7 (4.5)	2 (2.2)	2 (10.5)	11 (4.1)	N/A		
**A wish to end my life myself**	23 (14.8)	6 (6.5)	0 (0.0)	29 (10.9)	N/A		
**A wish for a doctor to help me end my life**	8 (5.2)	4 (4.3)	2 (10.5)	14 (5.2)	N/A		
**A wish for another professional or someone close to help me end my life**	5 (3.2)	3 (3.2)	0 (0.0)	8 (3.0)	N/A		
**I do not know**	4 (2.6)	10 (10.8)	3 (15.8)	17 (6.4)	N/A		
**Having made concrete plans / taken steps**							
**No**	104 (67.1)	93 (100.0)	19 (100.0)	216 (80.9)	N/A	N/A	N/A
**Yes, namely:**^**a**^	51 (32.9)	0 (0.0)	0 (0.0)	51 (19.1)	N/A		
**Decisions about end of life**^**b**^	24 (15.5)	N/A	N/A	24 (8.9)	N/A		
**Membership of interest group**^**c**^	14 (9.0)	N/A	N/A	14 (5.2)	N/A		
**Contact with a Caregiver**^**d**^	9 (5.8)	N/A	N/A	9 (3.4)	N/A		
**Searched for means to end one’s life**^**e**^	6 (3.9)	N/A	N/A	6 (2.2)	N/A		
**Searched for information**	3 (1.9)	N/A	N/A	3 (1.1)	N/A		
**Attempted suicide**	2 (1.3)	N/A	N/A	2 (0.7)	N/A		
**Other**	3 (1.9)	N/A	N/A	3 (1.1)	N/A		
**Having seriously considered attempting suicide in the past 12 months**							
**Never**	20 (12.9)	93 (100.0)	0 (0.0)	113 (42.3)	N/A	N/A	N/A
**Once in a while**	80 (51.6)	0 (0.0)	0 (0.0)	80 (30.0)	N/A		
**Now and then**	38 (24.5)	0 (0.0)	0 (0.0)	38 (14.2)	N/A		
**Often**	15 (9.7)	0 (0.0)	0 (0.0)	15 (5.6)	N/A		
**Very often**	2 (1.3)	0 (0.0)	0 (0.0)	2 (0.7)	N/A		
**Not willing to answer**	0 (0.0)	0 (0.0)	19 (100.0)	19 (7.1)	N/A		
**Having made a suicide attempt in the past 12 months**^**f**^							
**No**	125 (92.6)^g^	N/A	N/A	125 (46.8)	N/A	N/A	N/A
**Yes**	3 (2.2)	N/A	N/A	3 (1.1)	N/A		
**Not willing to answer**	7 (5.2)	N/A	N/A	7 (2.6)	N/A		
**Preference not to have to experience the future**^**h**^							
**Median (Q1-Q3)**	5 (5–6)	5 (3–6)	6 (4–7)	5 (4–6)	1 (1–2)	**0**.**000**	**0**.**001**
**Finding life worthwhile at this moment**							
**Yes**	67 (43.2)	56 (60.2)	8 (42.1)	131 (49.1)	20,568 (98.5)	**0**.**000**	**0**.**013**
**No**	88 (56.8)	37 (39.8)	11 (57.9)	136 (50.9)	315 (1.5)		
**Being weighed down by the burden of life**^**i**^							
**Median (Q1-Q3)**	5 (4–6)	4 (3–6)	5 (4.5–6)	5 (3–6)	1 (1–2)	**0**.**000**	**0**.**014**

Results are presented as N (%) unless “Median (Q1-Q3)” is reported

Percentages may not add up to 100% because of rounding

Medians are reported with 25th–75th percentiles

N/A = not applicable

Statistically significant results (*p* < 0.05) are in bold

^a^*N* = 61 because respondents could name more than one plan/step in answering the open question

^b^Includes wish or order “do not resuscitate”, wish for euthanasia in due time (in some cases recorded in an Advanced Euthanasia Directive), refraining from medical treatment (in some cases recorded in an advanced directive), having made a testament, having written down something considering end-of-life (not specified)

^c^Interest group concerning voluntary end of life

^d^Includes receiving treatment (psychological or psychiatric)

^e^Includes respondents who already obtained means to end their life

^f^*N* = 135 due to the fact that respondents who answered “never” or “not willing to answer” to the previous question (having seriously *considered* a suicide attempt) were not asked this question

^g^Percentages are based on *N* = 135

^h^7-point Likert scale ranging from 1 (“not at all”) to 7 (“very strong”). Respondents could also answer “I do not know”. Therefore, *N* = 151, *N* = 83, *N* = 16, *N* = 250, *N* = 989 (comparison group), respectively

^i^7-point Likert scale ranging from 1 (“not at all”) to 7 (“very strong”). Respondents could also answer “I do not know”. Therefore, *N* = 154, *N* = 90, *N* = 18, *N* = 262, *N* = 1014 (comparison group), respectively

In response to the question how long they had had the death wish, 18.7% of the group PDW-NSI reported having had it their whole lives, and 62.9% for a few years.

One third of the group with an active death wish had made concrete plans or taken steps regarding their death wish. Almost half of those reported plans/steps anticipating end-of-life decisions such as refraining from medical treatment or arranging an Advanced Euthanasia Directive (8.9% of the PDW-NSI group). A suicide attempt was reported by 0.7% of the PDW-NSI group.

When asked how they would describe their wish, a majority of the 155 respondents with an active death wish reported a wish for a natural death (*N* = 108). Thirty-six respondents (0.17% of the total response) indicated a desire to end their lives themselves or by EAS. Thirteen respondents reported a wish for assisted suicide by a health care professional or someone close, representing 0.06% of the total response.

Despite having a persistent death wish, almost half of the respondents in the PDW-NSI group (49.1%) confirmed that they considered their life worthwhile at that moment, which was significantly less than the percentage of respondents who answered the differentiation question with “no” (98.5%). When asked to indicate the intensity of their preference “not to experience the future” on a 7-point scale, 50% of the PDW-NSI group scored between 4 and 6, and 25% scored 6 or higher. Respondents who answered “no” to the differentiation question had significantly weaker preference not to experience the future: more than 50% scored no higher than 2.

Similar differences were found between the groups with an active and a passive death wish. Respondents with an active death wish had significantly higher scores on the preference *not* to experience the future, and significantly less frequently found life worthwhile, than respondents with a passive death wish.

## Discussion

Our study shows that 1.25% of a representative sample of adults aged 55 and older report having a persistent death wish without being severely ill, based on their own perception of health assessed with validated self-report health measures. Of our sample, we categorized 0.73% as having an active death wish, some because they indicated having seriously considered ending their lives, and others because they reported having made plans or taken steps to realize their death wish in the near or distant future. A minority of the group with an active death wish indicated a desire to actually end their lives (0.17% of our sample). A part of this group reported a wish for assistance in fulfilling this wish (0.06% of our sample).

With regard to respondent characteristics, we found that a persistent death wish in the absence of severe illness occurs not only in the oldest old. There was no significant overall difference in age distribution between the group with PDW-NSI and the respondents not identifying with the qualifications “seeing no future for oneself, longing for death, while not being severely ill” (based on the differentiation question). Previous studies report a higher overall prevalence of death wishes among older persons [17, 18, 27–29] and an increase with age [17, 18]. For example, a Dutch study found that 2.2% of persons aged 65 and above had a wish to die or no wish to live in the past week [27], and a Canadian nationally representative survey among adults aged 55 years and over indicated a prevalence of suicide ideation of 2.2% [29]. Furthermore, a study among a sample of 12,107 respondents from the general population of Europe, showed an increase of passive death wishes with age [17]. Of those aged 50–65, almost 5% had a passive death wish, while this rate was three times higher for those older than 75. However, these studies included persons with severe illness, including depression. Our selection of persons without severe illness may explain the lower percentages of both active and passive persistent death wishes among older persons in our study compared to previous studies. Furthermore, it may explain why our study did not show a significant overall difference in age distribution while previous studies with regard to the prevalence of death wishes among older persons report an increase with age. After all, the older people become the higher the chance of severe illness resulting in a negative answer to the differentiation question and not being included in the group PDW-NSI. On the other hand, people’s expectations based on their age may influence their answers about their health state and to the question if they consider themselves severely ill or not [30]. As a result, it is conceivable that older persons may not categorize health complaints and disabilities as “severe illness” while younger persons may do so when in the same health.

The group PDW-NSI reported considerable health problems and possible prevalence of mild (HADS-D 8–10) and moderate [11–15] depression. Comparison with the group with a negative response to the differentiation question indicated significantly worse health for the group PDW-NSI. The group we identified as having a persistent death wish without being severely ill can therefore not be characterized as a group of healthy older persons, but we cannot draw conclusions on the relation between health problems and the death wishes.

Our study shows ambiguity in death wishes of older adults without severe illness, confirming findings in previous qualitative studies among older persons with death wishes [31, 32]. For example, almost half of the group PDW-NSI (49.1%) indicated finding life worthwhile at this moment, and respondents with a persistent death wish did not always report a strong preference *not* to witness the future. These ambiguities, combined with the finding that a death wish does not necessarily signify a wish to actually end one’s own life, make tending to the needs of this group a challenge for physicians and other health care providers. This challenge was also described by Van Humbeeck et al. [6]. Their research among nurses showed that recognizing what is going on is complicated by the elusiveness of the phenomenon. Besides, the process of responding to the needs of the persons concerned was surrounded by ambiguity and uncertainty.

In the current debate, the dominant image of “completed life” or “tiredness of life” is that of healthy persons over the age of 75 who, overseeing their lives, reasonably decide they would prefer to die [33]. This image may not be well suited, as suggested by our findings that those with PDW-NSI reported considerable health problems, that the majority of the respondents with PDW-NSI were under the age of 75, and that a substantial minority of the group PDW-NSI (18.7%) reported having had a death wish their whole lives. Moreover, the death wishes of the respondents were often ambiguous, which indicates that they may not always reasonably decide they would prefer to die. The fact that our results challenge the dominant image has significant implications for public health policies in dealing with the needs of older adults with “completed life” or “tiredness of life”. Besides, our results draw attention to the fact that we should be careful using terms such as “completed life” or “tiredness of life”, to prevent wrongful connotations. The findings of our study do not substantiate the positive connotation of the term “completed life”. The term “completed life” might obscure the health problems and social and existential struggles some people are dealing with. While the term “tiredness of life” does not have this positive connotation, it is still not an accurate representation of these struggles, and therefore seems not befitting as well. Consequently, we propose that a more descriptive definition may be better suited and more representative of the actual death wish under study.

Death wishes were considered active when respondents had seriously considered ending their lives or when they had made plans or taken steps regarding their death wish. Looking at the answers of respondents who indicated having made plans or taken steps, it is noteworthy that a large part of the reported activities can be viewed as anticipating a self-chosen death in due time rather than leading to a self-chosen death in the near future. For example, becoming a member of an interest group regarding voluntary end of life, or writing down one’s wishes regarding medical treatment or end-of-life care, are not necessarily steps towards ending one’s life in the near future. This corresponds with the finding that the majority of respondents with an active death wish long for a natural death. Only 0.17% of our total sample had an active death wish resulting in a wish to end their lives, divided into 0.11% who had a wish to end their lives themselves and 0.06% who had a wish for assisted suicide.

These results indicate that the boundaries between active and passive death wishes, and also between death wishes and suicidal ideation are not clear-cut. The lines between these concepts are very thin and therefore difficult to draw.

Furthermore, these results indicate a considerable difference between opinions and actual experiences considering death wishes and EAS. Although a growing group of Dutch citizens is in favor of legal prescription drugs for older persons [12], the group of older persons who actually wish to end their lives is small. In addition, it is important to note that opinions may shift depending on the specificity of the question. For example, while 51% of Dutch citizens reported to be in favor of allowing the oldest old to obtain lethal prescription drugs at their own request from a physician to end their own lives, only 38% were in favor of this in a case describing the situation of a 86-year old with a wish to die in the absence of severe illness [12].

The main strength of our study is that it is the first to provide representative data on the prevalence of persistent death wishes among older adults without severe illness. Our large sample, combined with information about non-response, provides robust and representative data. Additional strengths are the analysis in three steps to identify the group with a persistent death wish without severe illness, and the detailed insight into the nature and ambiguity of these death wishes, existential issues and actions towards realization of the death wish.

Our study also has several limitations. First, selection bias is always possible in surveys and when using existing access panels. For example, people with a death wish and a desire to end their lives may be less inclined to participate in surveys. Our sample showed small but significant differences with non-respondents on almost all background variables. However, as unweighted and weighted percentages do not show large differences, we assume that these differences did not have a large impact on the prevalence results and the results presented in Tables 1, 2, and 3.

Second, the quantitative method has limitations for the conclusions that may be drawn from our results. The categorization of the persistence of death wishes was based on respondents’ own recall which may be influenced by their current mood. Moods may also have influenced the self-perceptions of health. For the respondents in the group indicating a wish to actively end their lives, we are unable to determine whether they would want this wish to be realized immediately or in due time. Moreover, there may be a difference between having this wish and ultimately being willing or able to take the final step to end one’s own life. Therefore, the percentages regarding the prevalence may be overestimated and need to be interpreted with caution.

## Conclusions

In the Netherlands and Belgium the question whether older adults with a persistent death wish without severe illness should be allowed to receive EAS is a topic of societal and political debate. The lack of robust knowledge on the prevalence of older adults with a persistent death wish without severe illness and on their characteristics, existential issues and the nature of their death wishes has been an obstacle for decision making on the issue. This study among a representative sample of 32,477 Dutch citizens aged 55 and older reveals that a small - but substantial on a population level - group of older adults have a persistent death wish in absence of severe illness. The majority of these older adults long for a natural death, and a wish for help from a caregiver or someone close to end one’s life is rare among the group with an active death wish (0.06% of our sample). The identified characteristics challenge the dominant “completed life” or “tiredness of life” image of healthy persons over the age of 75 who, overseeing their lives, reasonably decide they would prefer to die. Furthermore, the death wishes without severe illness are often ambiguous and do not necessarily signify a wish to end one’s life. It is of great importance to acknowledge these nuances and variety in the debate and in clinical practice, to be able to adequately recognize the persons involved and tailor to their needs.

Our findings raise many questions that need to be addressed to inform decision making on how to respond to the needs of this group. For instance, which characteristics are the most important independent and decisive predictors of PDW-NSI? And to what extent do the health problems contribute to the death wishes of persons with PDW-NSI, although they consider themselves not severely ill? What do people mean when stating they have a wish to die? How do death wishes of persons with PDW-NSI develop over the years? Future quantitative and qualitative (longitudinal) research is needed to answer these questions.

## Supplementary information

**Additional file 1.** Questionnaire items reported in this study.

**Additional file 2.** Additional methodological justification.

**Additional file 3.** Respondents and non-respondents.

## Data Availability

The datasets generated during and/or analysed during the current study are not publicly available due to the fact that the project analyses have not been completed but are available from the corresponding author on reasonable request.

## Abbreviations

EAS: Euthanasia or physician-assisted suicide
PDW-NSI: Group of persons with a persistent death wish and no severe illness
