# Supplementary material for: Prevalence and characteristics of older adults with a persistent death wish without severe illness: a large cross-sectional survey
Source: BMC Geriatr. 2020 Sep 17;20:342. doi: 10.1186/s12877-020-01735-0 (PMC7495831; doi:10.1186/s12877-020-01735-0)
Supplement: Supplementary file 1 — Additional file 1. Questionnaire items reported in this study. [file 12877_2020_1735_MOESM1_ESM.docx]

**Additional file 1: Questionnaire items reported in this study**

- Worldview^[[1]](#footnote-1)^

  *Every individual - religious or not - has a worldview. A worldview is the way you look at mankind and the world. A worldview affects how people experience important events in their lives. The following questions address your worldview. Below are the most common worldviews. Can you indicate which applies or apply to you?*
  Christian (Protestant)
  Christian (Roman Catholic)
  Muslim
  Jewish
  Humanist
  Atheist
  Agnostic
  Spiritual but not religious
  Buddhist
  Hindu
  Anthroposophic
  Esoteric
  Other
  No worldview
- Number of children

  *How many children do you have?*

None

1 child

2 children

3 children

4 children

5 children

More than 5

- Current health state VAS

*Please indicate on the scale below how good or how bad your overall health is at this moment.*

11 point (0-10) Likert scale: 0 = The worst health you can imagine; 10 = The best health you can imagine

- EQ-5D-5L

  *For each of the following questions, please choose the answer that best describes your health at this moment.

  To what extent do you have problems walking?*

  I have no problems in walking about
  I have slight problems in walking about
  I have moderate problems in walking about
  I have severe problems in walking about
  I am unable to walk about

*To what extent do you have problems washing or dressing yourself?*

I have no problems washing or dressing myself
I have slight problems washing or dressing myself
I have moderate problems washing or dressing myself
I have severe problems washing or dressing myself
I am unable to wash or dress myself

*To what extent do you have problems carrying out your usual activities? By usual activities we mean, for example, work, study, housework, family and leisure activities.*

I have no problems doing my usual activities
I have slight problems doing my usual activities
I have moderate problems doing my usual activities
I have severe problems doing my usual activities
I am unable to do my usual activities

*To what extent do you experience pain or discomfort?*

I have no pain or discomfort
I have slight pain or discomfort
I have moderate pain or discomfort
I have severe pain or discomfort
I have extreme pain or discomfort

*To what extent are you anxious or depressed?*

I am not anxious or depressed
I am slightly anxious or depressed
I am moderately anxious or depressed
I am severely anxious or depressed
I am extremely anxious or depressed

- HADS depression subscale
   *The following statements are about how you feel. Please indicate which answer best reflects how you have felt in the past week.

  I still enjoy the things I used to enjoy*

  Definitely as much
  Not quite so much
  Only a little
  Hardly at all

  *I can laugh and see the sunny side of things*

  As much as I always could
  Not quite so much now
  Definitely not so much now
  Not at all

*I feel cheerful*

Not at all
Not often
Sometimes
Most of the time

*I feel as if I am slowed down*

Nearly all the time
Very often
Sometimes
Not at all

*I have lost interest in my appearance*

Definitely
I don’t take as much care as I should
I may not take quite as much care
I take just as much care as ever

*I look forward with enjoyment to things*

As much as I ever did
Rather less than I used to
Definitely less than I used to
Hardly at all

*I can enjoy a good book or radio or TV program*

Often
Sometimes

Not often
Very seldom

- Life-threatening disease

*Have you ever had a life-threatening disease, or do you have a life-threatening disease at the moment?*

No
Yes, I did have a life-threatening disease once, but not anymore
Yes, I currently have a life-threatening disease, namely … *Open

- Number of current diseases

  *Please indicate for the diseases listed below whether you are affected by them at this moment. Multiple answers possible.*

  Joint conditions (e.g. arthritis, gout, rheumatism)
  Neck or back problems
  Bone decalcification (e.g. osteoporosis)
  Diabetes
  Tightness of the chest (e.g. COPD, asthma)
  Crohn’s disease
  MS/ALS
  Skin disease
  Thyroid disease
  Heart failure/heart disease
  Consequences of a cerebral infarction/brain haemorrhage

Dementia
Parkinson’s disease
Cancer
Psychological complaints (mood or anxiety problems, depression)
Other
None of these diseases

- Burden of current diseases

  *How much does this disease/do these diseases bother you? This refers to the disease(s) you indicated in the previous question.*

  10 point (1-10) Likert scale: 1 = Very little; 5 = Fairly bothersome; 10 = Very much
- Number of current complaints

  *Please indicate for each of the following medical complaints whether you experienced it in the past week. Multiple answers possible.*

Hearing problems or deafness, tinnitus
Eye problems and visual impairment
Memory problems
Difficulty speaking
Headache
Sleep problems
Falls (or fear of falling)
Problems walking
Dizziness
Problems with particular movements
Depression (depressive feelings)
Lack of appetite
Overweight, obesity
Incontinence (urinary or bowel)
Obstipation, hard/slow bowel movement
Impotence
Loss of sense of smell or taste
Chronic itching (for example due to dry skin)
Bedsores
Extreme/chronic fatigue
(Chronic) pain
Other
None of these complaints

- Burden of current complaints

  *How much does this complaint/do these complaints bother you? This refers to the complaint(s) you indicated in the previous question.*

10 point (1-10) Likert scale: 1 = Very little; 5 = Fairly bothersome; 10 = Very much

- Duration of having a death wish

*How long have you had a wish to be dead? Please choose the answer that best describes your situation*

Basically my entire life
For several years
About one year
About six months
About one month
About one week

- Characterization of the death wish

*People can long for death in different ways. Which of the following statements best describes you? Please indicate only one answer.*

A desire for a natural death that just happens
A desire to not wake up tomorrow and die in my sleep
I feel my current situation is unlivable

A wish to end my life myself
A wish for a doctor to help me end my life
A wish for another professional or someone close to help me end my life
I don’t know

- Having made concrete plans/taken steps

*Have you made any concrete plans or taken steps in this process? If so, which plans or steps?*

No, at the moment I have no concrete plans and I have not taken any steps
Yes, I have made concrete plans and/or taken steps, namely … *Open

- Having seriously considered attempting suicide in the past 12 months

  *In the past 12 months, have you ever seriously considered ending your life?*
  Never
  Once or twice
  Occasionally
  Often
  Very often
  I don’t want to answer this question
- Having made a suicide attempt in the past 12 months

*In the past 12 months, have you attempted to end your life?*

Yes

No

I don’t want to answer this question

- Preference not to have to experience the future

*Here is a statement about how you look at the future. Please indicate the degree to which you can identify with it.*
*I would prefer not to have to experience the future*
7 point (1-7) Likert scale: 1 = Not at all; 7 = Very strongly; and I don’t know

- Finding life worthwhile at this moment

Do you find life worth living at this moment?

Yes
No

- Being weighed down by the burden of life

Below you find a statement relating to your current situation. Again, we are interested in your own experiences in the past week. Please indicate the degree to which you can or cannot identify with it.

The burden of life weighs me down

7 point (1-7) Likert scale: 1 = Not at all; 7 = Very strongly; and I don’t know

1. Other background characteristics of the study sample were already known. [↑](#footnote-ref-1)
