# Supplementary material for: Prevalence and characteristics of older adults with a persistent death wish without severe illness: a large cross-sectional survey
Source: BMC Geriatr. 2020 Sep 17;20:342. doi: 10.1186/s12877-020-01735-0 (PMC7495831; doi:10.1186/s12877-020-01735-0)
Supplement: Supplementary file 2 — Additional file 2. Additional methodological justification. [file 12877_2020_1735_MOESM2_ESM.docx]

**Additional file 2: Additional methodological justification**

***Selection process to identify the PDW-NSI group (step 1 and 2)***

*Step 1 – No severe illness: self-reported health*

Two self-reported health measures were used for this first step in the selection process. First, we used respondent’s scores on a visual analogue scale (VAS), employing an 11-point scale ranging from 0 (worst imaginable health state) to 10 (best imaginable health state). In literature, a cut-off point to distinguish severe illness using VAS scales is not available. Informed by recommended cut-offs for severe pain we defined “no severe illness” as VAS $\geq$ 4 (1-3). Second, we used the EQ-5D-5L. The cut-off point was determined based on a scatter plot combining the VAS scores and EQ-5D-5L sum scores of our study sample, see **Figure 1**. As the regression line at the level of VAS = 4 crossed the EQ-5D-5L axe just before 17, we decided to interpret EQ-5D-5L sum scores $<$17 as “not severely ill”. For the scatter plot, see the Supplementary Appendix.

Combining these two cut-offs, respondents were categorized as “not severely ill” if they scored $\geq$4 on the VAS *and* $<$17 on the EQ-5D-5L (sum score). Respondents categorized as “severely ill” (VAS $<$4 *or* EQ-5D-5L sum score $\geq$17) were thus excluded in the selection of the group PDW-NSI.

**Figure 1: Scatterplot of VAS scores combined with EQ-5D-5L sum scores**

**
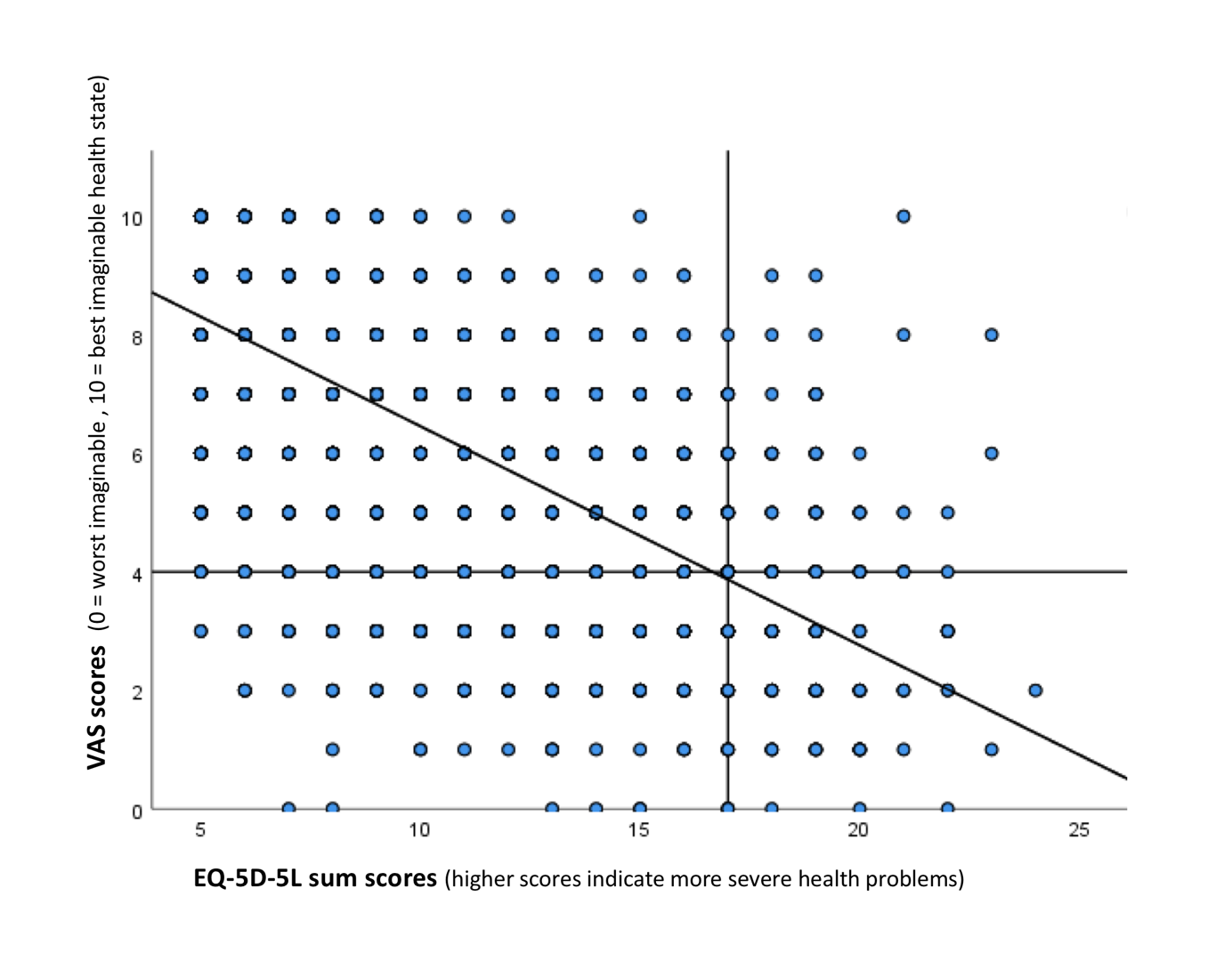
**

*Step 2 - No severe illness: indication for depression*

HADS-D sum scores were calculated using the first six items from the first round, combined with the seventh item of the second round. Test-retest reliability based on the first six items of the first round and second round was high (intraclass correlation coefficient 0.83). For missings in the second round, the “half-rule method” was applied, using the mean of the first six items to calculate the seventh item (4). The value of the seventh item was multiplied by 0.70, as the second round data showed that on average, the seventh item had been scored 0.70 times the mean of the first six items. To classify respondents into one of the four categories, the whole numbers before the decimal points of the sum scores were used.

Respondents with an indication for severe depression (HADS-D sum score $\geq$ 17) were excluded in the selection of the group PDW-NSI.

*Step 3 - Persistence of the death wish*

In compliance with literature about death wishes and suicidal feelings, we considered a death wish with a duration of $\geq$ 1 year as “persistent” (5). Respondents who recalled a duration of their death wish of less than one year were thus excluded in the selection of the group PDW-NSI.

**References**

1. Boonstra AM, Preuper HRS, Balk GA, Stewart RE. Cut-off points for mild, moderate, and severe pain on the visual analogue scale for pain in patients with chronic musculoskeletal pain. Pain®. 2014;155(12):2545-2550.

2. Loos MJA, Houterman S, Scheltinga MRM, Roumen RMH. Evaluating postherniorrhaphy groin pain: visual analogue or verbal rating scale? Hernia. 2008;12(2):147-151.

3. Serlin RC, Mendoza TR, Nakamura Y, Edwards KR, Cleeland CS. When is cancer painmild, moderate or severe? Grading pain severity by its interference with function. Pain. 1995;61(2):277-284.

4. Bell ML, Fairclough DL, Fiero MH, Butow PN. Handling missing items in the Hospital Anxiety and Depression Scale (HADS): a simulation study. BMC research notes. 2016;9(1):479.

5. Paykel E, Myers J, Lindenthal J, Tanner J. Suicidal feelings in the general population: a prevalence study. The British Journal of Psychiatry. 1974;124:460-9.
