# Supplementary material for: Prevalence and characteristics of older adults with a persistent death wish without severe illness: a large cross-sectional survey
Source: BMC Geriatr. 2020 Sep 17;20:342. doi: 10.1186/s12877-020-01735-0 (PMC7495831; doi:10.1186/s12877-020-01735-0)
Supplement: Supplementary file 3 — Additional file 3. Respondents and non-respondents. [file 12877_2020_1735_MOESM3_ESM.docx]

**File 3: Respondents and non-respondents**

**Table 1. Background characteristics of respondents and non-respondents**

| **Aspects and items** | | **Respondents N= 21294** | **Non-respondents  N= 11183** | **P-value**  Respondents  vs.  Non-respondents |
| --- | --- | --- | --- | --- |
| **Gender** | | | | |
|  | **Female** | 10731 (50.4) | 6090 (54.5) | **0.000** |
|  | **Male** | 10563 (49.6) | 5093 (45.5) |  |
| **Age (years)** | | | | |
|  | **Median (Q1-Q3)** | 65 (60-72) | 66 (60-73) | **0.000** |
|  | **55-59** | 5218 (24.5) | 2640 (23.6) |  |
|  | **60-64** | 4662 (21.9) | 2197 (19.6) |  |
|  | **65-69** | 4232 (19.9) | 1948 (17.4) |  |
|  | **70-74** | 4041 (19.0) | 2060 (18.4) |  |
|  | **75-79** | 1836 (8.6) | 1176 (10.5) |  |
|  | **80-84** | 913 (4.3) | 726 (6.5) |  |
|  | **85-89** | 332 (1.6) | 321 (2.9) |  |
|  | **90-94** | 54 (0.3) | 104 (0.9) |  |
|  | **95-99** | 6 (<0.1) | 11 (0.1) |  |
| **Educational attainment^a^** | | | | |
|  | **Low** | 7173 (33.7) | 4646 (41.5) | **0.000^b^** |
|  | **Middle** | 7714 (36.2) | 3775 (33.8) |  |
|  | **High** | 6305 (29.6) | 2626 (23.5) |  |
|  | **Unknown** | 102 (0.5) | 136 (1.2) |  |
| **Household size (number of persons)** | | | | |
|  | **1** | 5463 (25.7) | 2827 (25.3) | **0.046** |
|  | **2** | 12646 (59.4) | 6568 (58.7) |  |
|  | **3 or more** | 3185 (15.0) | 1788 (16.0) |  |
| **Social class^c^** | | | | |
|  | **Low** | 8625 (40.5) | 5289 (47.3) | **0.000** |
|  | **Middle** | 3238 (15.2) | 1677 (15.0) |  |
|  | **High** | 9431 (44.3) | 4217 (37.7) |  |
| **Urbanization^d^** | | | | |
|  | **Very high** | 3941 (18.5)^e^ | 2009 (18.0)^f^ | 0.267 |
|  | **High** | 7422 (34.9) | 3935 (35.2) |  |
|  | **Moderate** | 3883 (18.2) | 1993 (17.8) |  |
|  | **Low** | 4295 (20.2) | 2293 (20.5) |  |
|  | **None** | 1747 (8.2) | 950 (8.5) |  |

*Results are presented as N (%) unless “Median (Q1-Q3)” is reported.

*Percentages may not add up to 100% because of rounding.
*Medians are reported with 25^th^-75^th^ percentiles.
*Statistically significant results (p < 0.05) are in bold.

^a^ Low= lower vocational education, lower secondary education, or less; Middle= intermediate vocational education or higher secondary education; High= higher vocational education or university; Unknown= I do not know/want to answer.

^b^ N= 32239 because category “Unknown” was not included in the test.

^c^ Based on educational attainment and profession of the main breadwinner.

^d^ Very high= $>$2500; High= 1500-2500; Moderate= 1000-1500; Low= 500-1000; None= $<$500 addresses per km².

^e^ N= 21288 in this column due to 6 missings for this variable. Percentages are based on N=21288.

^f^ N= 11180 in this column due to 3 missings for this variable. Percentages are based on N=11180.
